# Collectanea

**Published:** 1827-09

**Authors:** 


### COLLECTANEA.

Floriferis ut apes in saltibus omnia libant,
Omnia nos, itidem, depascimur aurea dicta.

### MEDICINE.

*Case of unusual Constipation.*—The subject of the following case, (detailed by Dr. Crampton, in the *Dublin Hospital Reports*,) a slender, but originally healthy and active young lady, had scarlatina several years since, in so slight a form as scarcely to require confinement to bed, but her convalescence was slow, and was followed by a severe pectoral complaint: this she contracted in her exertions to attend the other members of her family. She became emaciated, and there was every reason to apprehend the rapid advance of phthisis. The medical treatment did little more than mitigate or arrest the progress of the symptoms: indeed, consumption seemed inevitable. She was removed to Mallow, where she spent the winter and spring. A considerable amelioration was the result of this measure, but still she was completely a valetudinarian, retaining her emaciated appearance. Her cough continued, attended with a heavy expectoration, and aphthæ were occasionally seen in the mouth and fauces; the tongue was sore and red, and not unfrequently a troublesome diarrhœa occurred; the catamenia continued regular. She seldom left the house, and in cold weather rarely even her chamber. She required constant medical guidance, so that few days elapsed without my seeing her. In this way her health continued nearly stationary for about seven years, except that every now and then, on catching fresh cold, she suffered a temporary aggravation of her pulmonic disorder.

In February 1819, during a short absence of mine from Dublin, she was attacked with a severe diarrhœa, attended with considerable pain. For this she would take no remedy, as her physician, who alone was supposed to know her constitution, was absent. This diarrhœa, which probably was attended with inflammation of the mucous membrane of the bowels, ceased after some days, but was exchanged for a costive state, accompanied with considerable pain in the abdomen. In fact, peritoneal inflammation, first in a chronic, afterward in a subacute form, appeared to have been established, and on my return to town I had the mortification to see how little had been done, and how much time had been lost in a case of great urgency. Her illness now assumed such a threatening aspect, that I thought every night would have put a termination to her sufferings. Discharges from the bowels were rarely procured, and these with considerable difficulty; but, though at this time no absolute obstruction appeared to exist, there was reason to apprehend that extensive adhesions had taken place between the convolutions of the intestines. She took little sustenance, rejecting almost every thing from her stomach, and being emaciated to the last degree, altogether confined to bed, and unable to assist herself. There was every prospect at this juncture that death must soon put an end to her miseries. The result, however, turned out otherwise. The symptoms remained stationary for a considerable time, and circumstances made it necessary that her family should remove to about fifty miles from Dublin. As the canal extended near their new residence, I advised that she should be taken from her bed, and carried on a mattress to the boat, where she was placed in a bed, and she actually bore the journey well, to the surprise of her family. After she was settled in the country, she continued to keep her bed, the abdomen was tender and inflated, she seldom had a stool more than about once a week; all medicines were rejected from the stomach; the greater part of her sustenance, which was all liquid, shared the same fate; what she brought up was offensive in the extreme, and often evidently stercoraceous; in some instances it had a urinous taste and smell, little urine being either secreted into or passed from the bladder. Notwithstanding the uncomfortable condition in which she existed, after a little time it became evident that she bore her distress and pains with more facility. The symptomatic feverish state subsided, the pulse became natural; she took liquid food in sufficient quantity; there was less emaciation; the vomiting was less severe, but she threw up every day, and the intervals between the times of emptying the bowels became longer; the urine was seldom passed, and then with pain, and in very small quantity; the catamenia were still regular. She even appeared to have regained a little flesh, and slept well. Her usual sustenance was tea, toast, milk, and gruel, but no solid food: no swelling now, or fulness in the abdominal region; she has either lost the use of her lower extremities, or is averse to use them.

She is now in her thirty-seventh year, and has been in the state described for the last seven years. For the last eight months she has had no passage from her bowels, and only two or three during the preceding year, and she scarcely passes any urine. There is no possibility that any deceit can be practised, as she sleeps in a room with a confidential attendant, and is watched closely by her parents and her sister. She never makes the slightest locomotive exertions with her lower limbs, but has the complete muscular power of the upper extremities. She is carried every day into the drawing-room after she has taken her breakfast, placed on a sofa, where she remains until she is again replaced in bed for the night. Her disposition, which formerly was cheerful and gentle, has become peevish and irritable. It is quite impossible to induce her to comply with any direction or measure which is proposed for her relief. She takes no medicines; she makes no effort to amuse herself either with work, books, or music, although formerly much engaged in such occupations. She never appears to have had any apprehension of danger, but always to have entertained hopes of recovery, as phthisical patients are known to do.

I recollect a case somewhat resembling this in a patient named Ann Free, who was under my care for a considerable time in Steevens's Hospital, but I have lost the notes of the case. In her both the bowel and urinary discharges were nearly suppressed. When she had a stool it was considered quite an extraordinary occurrence, and she never passed urine except when relieved by the catheter. She vomited occasionally excremental matter; and, for a considerable time, when no urine appeared to be secreted, she threw up a fluid of a urinous taste and smell. She lived for several years. By persevering with injections at proper intervals, the bowels were cleared, and the constant use of the catheter seemed to restore in some degree the secretion of urine, but she ultimately sunk under the pressure of disease in the bladder. After death the colon was found immensely distended, whilst a lower portion of that intestine was contracted to so small a dimension as to amount to a stricture scarcely pervious. The bladder was thickened, and otherwise diseased in structure.

---

*The Propagation of Inflammation by Contiguity*—is well illustrated in the following case, taken from the Clinical Report of the Meath Hospital. In the dissection of a fatal case of enteritis, we observed that the omentum, which lay extensively over the intestines, was healthy, except where it was in contact with the inflamed portions of the intestines. These portions were circumscribed and limited in extent, some highly vascular and red, others in a gangrenous state, and one actually perforated. The perforation was small, not exceeding a line in diameter. The inflamed portions of the omentum were very vascular and red, about the size of a dollar, and *lay exactly over the inflamed portions of the intestine.* It is to this correspondence in their situation with the inflamed parts of the intestine, that we wish to direct the attention of our readers. Similar facts have been frequently observed in diseases, but, as far as we know, they have never been satisfactorily explained. Thus, when a portion of the pleura pulmonalis is much inflamed, a portion of the pleura costalis corresponding, or opposite to it, is always found to be also inflamed. When, on the other hand, inflammation spreads from the intercostal muscles to the lungs, as in the conversion of pleurodyne into pneumonia, it does not traverse the pleura, in order to reach the lungs, by following the reflection of this membrane over them, but passes at once, and directly from the pleura of the ribs, to that of the lungs, between which there was previously no direct communication. To this circumstance is owing the adhesions between serous membranes, so readily formed by inflammation; for, when a part of one becomes inflamed, the portion of the other in contact with the inflamed parts assumes also an inflammatory action, and, lymph being thrown out by both, a false membrane is finally formed, not less intimately connected with one than with the other.

In enteritis we have repeatedly observed that the peritoneum lining the abdominal parietes is most inflamed in those parts which had been in contact with the most inflamed portions of the intestines. Another very important analogous fact was observed by Dr. Wilson Philip, in his Treatise on Indigestion. In the second stage of that disease, the pylorus frequently becomes inflamed, producing tenderness in the epigastrium, (page 105 ;) and as the pylorus, he observes, "lies with the thin edge of the liver upon and in contact with it, the inflammatory process is communicated to the thin edge of the liver, and thus inflammation of the liver is occasioned, and terminates in evident enlargement and tenderness of that organ."

We venture to propose the following explanation of these facts. When a portion of a serous membrane becomes inflamed, it is rendered highly vascular; it becomes at first dry and rough, but afterwards exhales either a morbid fluid secretion, or coagulable lymph; there is some reason to believe that its temperature is also increased. Now, in this state of things, that portion of the opposite membrane which corresponds to it is thus exposed to the contact of a membrane whose *sensible properties* are altogether altered from their natural state, and which may therefore be now considered to be as it were a *foreign body*, which, presenting a surface quite different from that to which the sensibility of the opposite membrane had been accustomed, must of course act as a stimulus to it, and thereby excite in it an inflammatory action. This explanation seems at least more satisfactory than Mr. Hunter's sympathy of contiguity.

---

*Case of Arachnitis, treated with Quina and Opium.*—Our readers will probably be rather startled at the title of this article, but let the case speak for itself. A woman, named Vignaux, aged twenty-eight, of nervous temperament, suckling her first child in the third month, experienced, towards the latter part of autumn, violent pains in her head. Venesection was recommended, but she would not submit to it. Continued fever, with obscure remissions, came on. The bleeding, which was now practised, neither repressed nor even moderated these symptoms, but, on the contrary, seemed to aggravate them. When M. DESCRIMUS first saw the patient, she had the following symptoms:—The eyes fixed; tongue clean and moist; pulse weak and unequal; heat below the natural standard; respiration almost imperceptible; limbs stiff; the belly much sunk. The features of the face alone showed signs of life. An antispasmodic potion was recommended, and cold affusions on the arms, if convulsions should again supervene. These did accordingly come on, and continued till the next day. To the furious delirium of the night, a comparative quiet had succeeded, which was in turn replaced by the symptoms of the foregoing night. These accessions had now been reproduced during three successive nights, when it was decided to try the Quina, having previously had the sanction of Dr. CASTEX's advice. The patient took the following mixture by spoonsful:—℞. S. Quinæ gr. x.; Syrup. Quinæ ℥ ij.; Syrup Dincad. ℥ j.; Aquæ ℥ iij. M. The next night the convulsions and delirium were less, but the intellectual derangement continued. The medicine was repeated, as well as occasionally a dose of two ounces of oil of sweet almonds. These means soon produced a decided change, which showed itself in a copious alvine evacuation. The paroxysms were dissipated, and there only remained a trifling degree of fever, and still a little derangement of the intellect, which continued two months.

We regard this case (taken from one of the French Journals,) as a good illustration of the loose manner in which our Gallic neighbours apply the term *Arachnitis*. There is no evidence whatever either of the arachnoid, or any other membrane of the brain, having been inflamed.

---

### SURGERY.

*A Case of Inflammation of the Tongue cured by Incisions*—is detailed by Mr. CHARLES MARTIN in the last Number of the Edinburgh Journal.—A stout healthy man, aged thirty-five, came, on the 9th of February last, to consult me about the state of his tongue. On interrogating him, he gave me the following particulars:—

On the 7th he was employed in building the wall of a house. Not long after he returned home in the evening from his work, he felt an acute pain in the tongue, at first so circumscribed that it could be covered with the point of the forefinger, but speedily extending over the whole surface of the organ. The pain was attended with a distressing sensation of heat, and a feeling as if the tongue was gradually and uniformly enlarging. The lingual arteries throbbed violently, and the saliva adhered tenaciously to the roof of the mouth and surface of the tongue. He was restless and a little feverish, and complained of head-ache. With little variation in the symptoms, the tongue continued to increase to such a size that it protruded from the mouth, separating the jaws to the extent at least of half an inch. When the disease assumed this aspect, he became dreadfully alarmed, and between twelve and two o'clock called upon me.

On examining the tongue, I found that it occupied a large proportion of the cavity of the mouth; and I could with difficulty introduce my little finger between it and the upper jaw. It felt smooth and hard to the touch, and had a thick coating of viscid mucus. From the high degree of tension, its point presented a glistening appearance. The patient had his jaws wrapped up in flannel; his countenance betrayed great anxiety; his pulse beat strong and frequent, and his skin was hot.

The treatment adopted was as follows:—

I opened a vein at the bend of the arm, and withdrew blood to the extent of thirty ounces. Having secured the wound, I remained a few minutes to observe the result of the bleeding, and before I quitted the apartment he thought himself rather relieved. In an hour afterwards I returned, and repeated the venesection to the same extent, which produced good effects, for he could articulate somewhat intelligibly. I had hopes that the swelling would gradually decrease without further interference with the lancet; but, if not, I was decided upon the steps I should next pursue.

Three hours afterwards, I repeated my visit; but instead of finding, as I expected, evident marks of improvement, the disease, on the contrary, had rapidly increased. At this period respiration through the mouth was totally suspended; and he could not breathe even through the nostrils but with difficulty. His countenance was flushed and anxious; the pulse was fluttering; his breath offensive. In short, he was threatened with immediate suffocation.

It was now perfectly obvious that the only remedy by which the patient's life could be saved was the knife. His head being properly secured, and the mouth opened as wide as possible, a scalpel was introduced with its flat surface along the tongue, and a deep incision was made in the most prominent part of the right side. On withdrawing the scalpel, a quantity of blood, mixed with *puriform* matter, issued from the wound. In a very short time he felt relieved. When this wound ceased to discharge blood, I made other two incisions, one in the middle, and the other in the left side of the tongue, but no purulent matter followed. The bleeding was promoted by gargling the mouth with warm water.

This local treatment produced such a happy and immediate effect, that, in a quarter of an hour after the first incision was made, the patient could articulate words pretty distinctly; his respiration became free and easy, and his countenance lost its anxiety. The joy he felt at this sudden and favourable change was extreme. He was, to use his own terms, like a man unexpectedly snatched from the grave.

On the following morning I paid him a visit. I found him sitting on the edge of the bed quite cheerful and happy. I left him with strict injunctions that he should avoid exposure to cold, and gave him a few purgative medicines. In a few hours thereafter he was walking in the street with his head fortified with flannel.

*Remarks.*—In conclusion, it may not be altogether nugatory to offer a very few observations upon the preceding case.

1. That the disease was inflammation, and that violent in its symptoms, and consequently rapid in its progress, is evident from the fact that purulent matter was largely secreted. Previous to the first incision being made, I could not possibly determine, either from existing symptoms or by external examination, whether or not there was a collection of pus. Could I have satisfied myself of this, I would not have prolonged the patient's sufferings by employing the lancet, but would have had immediate recourse to the knife. As the matter was deeply lodged in the substance of the tongue, I remained ignorant of its formation until the scalpel was used.

2. It is hardly necessary to remind the reader that, to effect any real and permanent good, the incisions should be made deep and extensive: otherwise he is only torturing his patient with pain, likely to prove more injurious than beneficial. The operation is exceedingly simple, and is not attended with the smallest danger, so that the surgeon may safely act with boldness and promptitude. Indeed, such is the urgency of the case that it imperatively demands vigorous interference; and trifling remedies are only dangerous instruments in the hands of a surgeon. In the case related above, it is questionable if the man could have survived for any length of time, unless the enlargement had been subdued by the timely use of the knife; for the pus was so deeply seated that he might have been suffocated before it could make its way outwards.

3. It may be observed, that, if this disease is early combated by copious depletion with the lancet, it may, as in other cases of inflammation, be speedily cured; but, when this treatment proves ineffectual, I know of no other remedy by which the patient's life may be saved except incisions.

4. And lastly, I have been induced to lay the preceding case before the public, because it is one, among many others, which sanctions the propriety, and illustrates the efficacy of having recourse to the scalpel, when general blood-letting fails to produce the desired effect. (*Edinburgh Med. and Surg. Journal.*)

---

*Amputation of Portions of the Lower Jaw.*—The following interesting paper, by Dr. Cusack, is from the *Dublin Hospital Reports.*—The animal structure of bone throughout the whole osseous system is liable to become the seat of certain morbid growths, which occasion different degrees of suffering and danger, according as they vary in magnitude, character, or situation. Such tumors frequently occur in the maxillary bones; causing, from the peculiarities of their situation, not only great deformity and distress, but, in many instances, the death of the patient.

These morbid growths are remarkable for having their origin in the medullary structure of the bone; to attempt, therefore, the removal of the disease by the knife, cautery, or other means, has been found not only ineffectual, but injurious; any violence thus offered to the part being uniformly followed by an increased rapidity of growth and aggravation of all the symptoms.

Amputation or excision of the entire of that portion of bone in which the disease has its origin, is the only measure that can be relied upon to ensure success; and this operation, although often perhaps impracticable in the superior maxilla, may be performed with more ease and safety when the disorder is situated in the lower jaw, than could have been at first imagined.

Those diseases which require amputation of the inferior maxilla, are either cancerous affections, commencing in the soft parts and contaminating the bone by their continuity; or morbid growths originating in the medullary structure, and endangering life by their effects upon the system generally, or their interference with the functions of the neighbouring organs.

In true cancer, commencing in the lips or cheek, and secondarily affecting the bone, the uncertainty of being able to extirpate every germ of the disease, and thus secure the patient against a return of the complaint, forms a serious objection against the employment of this operation. A similar objection, however, does not apply in those cases popularly denominated fungous tumors of the maxillary bones. In such instances the disease is more purely local, being primarily confined to the osseous texture, and incapable of contaminating the adjacent soft parts, or of affecting the system through the medium of absorption.

Effectually to extirpate the diseased structure, and secure the patient against a return of the complaint, amputation of a part or the entire of the jaw can alone be relied upon; and this operation, although so formidable in appearance, and often rendered complex and tedious by the situation or extent of the disease, may in general be performed with success and safety; even the excision of the bone from the articulation being attended with less difficulty or danger than could have been at first anticipated.

Amputation of the lower jaw has been but seldom performed in this country, although the operation is not uncommon upon the continent: in one instance only, as shall be hereafter noticed, does the removal of the bone from the articulation appear to have been attempted; and of this case no particulars, so far as I can recollect, have been recorded in any of our journals.

Before we can duly appreciate the advantages to be derived from amputation of the inferior maxilla, we must contrast the hideous deformity and cruel sufferings consequent upon the presence of tumors in this situation, with the comfort and safety secured to the patient by the performance of this operation. The loss of even large portions of the lower jaw is, in general, attended with but little inconvenience, and scarcely any perceptible deformity; while the extirpation of the original seat of disease is the best security against any return of the complaint.

Case I.—Honora Doyle, ætat. forty-six, a country-woman, of a healthy constitution, was admitted into the hospital, June 21st, 1824. She gave the following account of the commencement and progress of her complaint. In May, 1818, she found it necessary to have the second molar tooth of the left side of the inferior maxillary bone extracted: about six weeks afterwards she received a severe blow on her jaw, exactly over the situation which the tooth had occupied. This injury was not immediately followed by any unpleasant consequences, and was almost forgotten, when, in the succeeding September, her attention was excited by the appearance of a small tumor about the size of a hazel-nut, which protruded from the vacant alveolar space. The swelling, when first perceived, was firm, elastic to the touch, and almost insensible to pressure. According to her statement, it was unattended, in its incipient state, with any painful sensation; its progress was at first so slow as to be almost imperceptible, but in some weeks the swelling had become visible externally. She then applied for assistance to an apothecary, who pushed a lancet into its centre: this wound produced a profuse hemorrhage, which was suppressed with difficulty. She subsequently made application to other practitioners, by some of whom incisions were made, which were followed by similar results; she also stated that, after each incision, the tumor evinced a greater disposition to enlarge. At the expiration of the first year, the tumor had engaged a large portion of the bone, advancing towards the chin, and expanding inwards, so as to impede the motions of the tongue and prevent mastication. Still she was free from pain, and experienced no inconvenience except what depended on the size and situation of the swelling.

About this time she again applied to a neighbouring surgeon, who made a very deep incision into the centre of the tumor; this operation was followed by an immediate and rapid extension of the disease. Discouraged by these repeated failures, no further application was made for relief until the rapidly increasing size of the swelling, which now impeded respiration and deglutition, combined with the distress occasioned by profuse ptyalism and occasional hemorrhages, induced her to come to town, with the determination of submitting to the performance of any operation that might be proposed.

When she presented herself at the hospital, I found almost the entire of the left side of the lower jaw involved in the disease; the tumor projected outwards, and caused much deformity, the jaws being separated by a portion which protruded between the teeth, and prevented the closure of the lips. On inspecting the cavity of the mouth, the tumor appeared to consist of three branches, involving the bone in their centre; the first, or outer, formed the prominence which was visible externally; the second, ascending between the upper jaw and cheek, and distorting the countenance, reached as high as the margin of the orbit; the third, filling up the sublingual cavity, elevated the tongue, and pushed it to the opposite side: this portion likewise extended so far backwards as to press on the anterior arch of the palate. The teeth were observed on the surface of the tumor, sunk into its substance, and perfectly moveable. The entire portion of the jaw, included between its angle on the left side and the last incisor tooth on the right, was more or less affected.

The situation and extent of the disease will explain the nature of her sufferings. She complained of difficulty of respiration and deglutition, articulated indistinctly, and was unable to take nourishment, except in a liquid state. A continued discharge of saliva, mixed with bloody sanies, streamed over that portion of the tumor which protruded from her lips. Her general health, however, might be considered good: she had not yet suffered much constitutional disturbance, and preserved a tolerable appetite. Under these circumstances, the rapid increase of the disease threatening soon to put a period to her existence, it was decided, in consultation, to attempt the removal of the diseased portion of bone, as the only measure that afforded a prospect of saving the patient's life.

On Friday, July 7th, I proceeded to the operation, in presence of the surgeons of the hospital, Messrs. Crampton, Peile, Colles, and Wilmot. The patient was seated on a chair, her head supported, and inclined to the left side; this position being deemed preferable to the recumbent posture, as best calculated to favour the escape of the blood, and prevent its accumulation in the fauces.

Standing before the patient, I commenced by making an incision from the commissure of the lips, on the right side, which, passing obliquely downwards, and dividing the parts completely through, terminated about half an inch below the base of the jaw; the bone being thus laid bare, was divided by a small hand-saw through the alveolar process of the right canine tooth, which had been previously extracted. The next incision extended from the lobe of the ear, in the direction of the ramus, to the angle of the jaw; and both were connected by a third, carried parallel to the base. Dissecting up the flap thus formed, I divided the masseter muscle, which was expanded over the anterior surface of the tumor; and denuded the bone midway between its angle and condyle. A needle, to which the chain-saw had been connected, was passed behind the ramus, the point being kept close to the bone; the saw moved with so much ease and freedom, that the patient did not appear sensible of the division of the nerve. I then pressed the tumor downwards, to put the soft parts (attached to the bone internally) on the stretch; and concluded the operation by cutting across the muscles connected with the base of the jaw, exactly at their point of insertion.

The hemorrhage was inconsiderable; the dental, and some branches of the facial artery, were secured by ligatures; dossils of lint were placed in the cavity, to give support to the cheek; and the divided parts brought into apposition, and retained by points of interrupted suture. A light dressing was applied, and the entire supported by a bandage and compress.

The operation was tedious, yet the patient appeared so little exhausted by its duration, or the loss of blood, that she was able immediately after to walk to her bed. She swallowed drink in small quantities, which was conveyed to the base of the tongue by means of a gum elastic tube, attached to the spout of a teapot.

In the evening (six hours after the operation,) her face was flushed, her cheek hot, and slightly swollen; but she suffered no constitutional disturbance, and appeared disposed to sleep.

Saturday.—She had slept during the preceding night, but was greatly annoyed by a profuse ptyalism, and complained of a feeling of tension in the cheek ; in other respects she seemed in a comfortable state. Having removed the bandage and compress, I directed the application of the saturnine lotion, and the administration of a cathartic enema.

From this date her recovery proceeded rapidly: when suppuration was established, the dossils of lint were withdrawn through the mouth, and the wound acquired, each successive day, a more favourable appearance. Complete union of the divided soft parts followed, and on the twelfth day she was able to walk about the ward. Her articulation became gradually more distinct; she took every kind of nourishment which did not require mastication; the ptyalism, however, still remained. The natural form of the mouth had been so much altered, first by the protrusion of the tumor, and subsequently by the loss of support from the chin, that she was obliged to afford artificial assistance, by means of a bandage, to the lower lip. This inconvenience diminished daily during her stay in the hospital, and scarcely any deformity was ultimately observable. She sometimes complained of the mobility of the remaining portion of the jaw, which was drawn by the action of the muscles within the line of the upper teeth, so as to require the assistance of her finger to replace it.

In six weeks after her arrival in town, she returned to the country in good health; and, according to the last account which I heard from her, still continues free from any disposition to a recurrence of the disease.

[Two other cases of a similar nature are detailed.]

*Amputation at the Articulation.*—The success which had attended our former operations suggested the possibility of extirpating the jaw at the articulation.

The practicability of this measure was fully discussed, and the anatomical relations of the joint carefully considered. The danger of hemorrhage appeared to present the only serious objection against the undertaking; the proximity of the ramus of the jaw to the termination of the external carotid artery, and the near relation of the internal maxillary to the articulation, seeming to render it impossible to disengage the joint without wounding one of these vessels.

A little careful examination, however, will show that neither of these arteries is in immediate contact with the jaw. The internal maxillary, which would appear more exposed to danger, inclines backwards in its passage behind the neck of the condyle, being distant about a quarter of an inch from the bone; the natural structure of the joint allows this distance between the artery and articulation to be still farther increased; so that, by sawing the bone through at any point, and separating the attachment of the temporal muscle, the capsular ligament may be opened anteriorly, the condyle dislocated, and the jaw disengaged, without endangering any vessel of consequence.

From the detail of the following cases it will appear that exarticulation is not only practicable, but not necessarily attended by any danger of formidable hemorrhage, from the proximity of the carotid or its branches. The preliminary step, therefore, of securing this artery in amputation of the jaw, as practised by Dr. Mott, must be regarded as rendering the operation unnecessarily complex.

CASE IV.—James Heron, ætat. thirty, of a good constitution, was received into the hospital, May 6, 1825. Some months previous to his admission, he felt acute pain in the last molar tooth of the left side of the lower jaw. The pain was so fixed, and so like a common tooth-ache, that he had the tooth extracted. A short time after its removal, he observed a small tumor emerging from the vacant space. The progress of this tumor was slow, but it continued to extend itself outwards, so as to become prominent in the cheek; and inwards, so as to displace the tongue and press it to the opposite side: six months only had elapsed from the extraction of the tooth until his arrival in town. I found the tumor situated immediately in front of the angle of the jaw, the bony prominence of which it had completely obscured. Superiorly, it extended beyond the zygoma; internally, it occupied one half of the sublingual space. The bone was enlarged, and the teeth loosened as far as the first incisor tooth on the same side. He complained of the inconveniences resulting from the interruption to deglutition and articulation; but was free from any other distressing symptom, except a pain occasionally felt in the centre of the bone, shooting upwards to the ear. It was evident that the angle and ascending branch of the bone were the parts principally engaged, and that the extent of the disease would probably require the removal of the bone at the articulation.

On Friday, May 13, the patient being seated on a high chair, in the position already described, I commenced the operation by an incision, extending from the commissure of the lips of the affected side to the base of the bone, which was divided at the second incisor tooth: another incision, beginning at the zygoma, was carried down over the articulation and in front of the ramus, terminating at the angle. The third, connecting the two former, passed obliquely upwards and outwards, from the termination of the first. I then dissected the cheek from the anterior surface of the tumor, which was obscured by the expansion of the masseter muscle. This being divided, the extent of the disease could be more readily ascertained. A portion of the tumor, ascending under the zygoma, completely filled up the space beneath the arch; and, from its size and position, retained the processes immoveably fixed, preventing the operator from using the jaw as a lever in the dislocation of the condyle. Under these circumstances, I decided on cutting across the ramus above the angle.

Having accomplished this object, I removed the section of bone included between the two divisions, with the corresponding part of the tumor. As the room thus obtained enabled me to ascertain that the coronoid process was partly absorbed and distinctly separated from the condyle, I directed my attention exclusively to the articulation.

Mr. Colles, seizing the extremity of the ramus in a strong pair of forceps, pressed the condyle against the anterior part of the capsular ligament: by this means the joint was penetrated with more safety and facility. I next enlarged the opening with a blunt-pointed bistoury, sufficiently to allow the protrusion of the head of the bone and the separation of its remaining connexions with the capsule, as well as the division of the attachment of the external pterygoid muscle. The operation was concluded by the removal of that portion of tumor which was seated beneath the zygoma.

It is remarkable that no vessel except the facial artery required a ligature, the hemorrhage from all the others ceasing immediately after their division. Small dossils of lint were placed in the cavity to give support to the cheek, which was then replaced, and retained in its situation by three points of interrupted suture: the inflammation which succeeded the operation was comparatively trifling, the external wound healing by the first intention. A small abscess, which formed in the vicinity of the glenoid cavity, was the only circumstance that occurred to retard the patient's recovery.

[Three similar cases are given: one only proved fatal, and that not so much from the operation as from the unfortunate supervention of erysipelas.]

---

*Case of Cancer of the Tongue.*—In a recent Number of the Revue Medicale, we find an account of extirpation of cancer from the tongue, in such a manner as to preserve the organ itself.

M. Thure, ætat. thirty-five, of good constitution, came into the hospital La Pitié, under the care of M. Lisfranc, in the beginning of September, 1826, affected with cancer of the tongue, occupying two-thirds of the right side of that organ, from its point to the base. The disease had existed since 1823; had begun by ulceration situated nearly an inch from its anterior extremity. The pains, slight at first and intermittent, became, at the beginning of 1826, continued and lancinating. Mastication was almost impossible, and articulation very difficult. The tongue was swollen, hard, and covered with ulcerations. On the right side, under the os maxillaris inferior, there were several lymphatic glands enlarged, and forming considerable swellings. The patient had been some time before on an antisyphilitic regimen, which aggravated the disease. Before coming into hospital, he had consulted several surgeons, who all thought that the tongue was affected throughout its whole thickness, and recommended the extirpation of the part affected. M. Lisfranc, having reduced the submaxillary swellings by leeches, agreed with M. Mayor, of Lausanne, to employ for the removal of the disease the plan by ligature, recommended by this distinguished practitioner.

September 20th, the operation was performed in the following manner:—The patient seated in a chair, the head a little bent forward, and well supported, a straight bistoury was carried along the inferior part of the tongue to the place where it ceased to be adherent to the subjacent parts. The instrument traversed the organ from below upwards, and from before backwards, so that its point came out near the epiglottis, where the disease terminated. A section from behind forwards was made to separate the affected part from the surrounding tissues, which it was intended to preserve. This part of the operation being completed, the cancerous tumor was included in the noose of a ligature to strangle its base. The patient did not suffer much, and lost very little blood. He was again put to bed, and had emollient drinks and detergent gargles.

21st.—The patient complained of severe pain; there was no diminution in size of the constricted part; there was an abundant salivation. The ligature was tightened, and the pains very speedily disappeared.

22d.—No change in the size of the tongue, but, what is remarkable, a cicatrix was formed between the sound and diseased parts, throughout the whole extent of the longitudinal incision which had separated them. This adhesion was immediately destroyed, and a piece of fine linen introduced to prevent its recurrence. The constricted part, deprived of nourishment, soon became black; all the cancerous points were converted into eschars.

The ligature was removed on the sixth day. To the astonishment of every one, on the seventh day, when the dead parts had separated, it was perceived that almost the whole of the tongue was preserved, the surface only of the organ having been diseased. By the aid of detersives and emollients, and a little cauterisation, the wounds soon healed, and the patient was dismissed.

Some months afterwards, M. Lisfranc presented him to the Academy: he then had had no relapse, and the only difference that he felt was his speech being not quite so free as before the disease.

This case is regarded by the French surgeon as remarkable in more points than one. It proves that lymphatic swellings attending cancer may be destroyed by the use of leeches; an immense advantage, which will render operations practicable that otherwise would not be so. It proves also the efficacy of the measure proposed by M. Mayor for removing such tumors by ligature. But by far the most important point is, that we see cases where apparently the whole organ is affected with cancer, when in fact it is only on the surface. This fact furnishes a new proof of the importance of M. Lisfranc's idea, that, before deciding to remove an organ that appears to be entirely cancerous, we should, by means of cautious incisions, discover its depth, and limit ourselves to the removal of such parts only as are affected.

---

"*Receipt for making a Rupture.*"—There is a kind of free-masonry among soldiers, which is perhaps conducive to the harmony of the barrack-room, but which, preventing the exemplary from exposing the worthless, and holding up the informer as an object of universal detestation, renders it extremely difficult to come to a right understanding of the various methods of simulating disease. I have no doubt that these methods have been systematised, and that they are preserved in many regiments, and handed down for the benefit of those who may be inclined to make trial of them. That this opinion may not appear fanciful, I shall, before proceeding further, relate one or two instances of systematic fraud practised for the purpose of deceiving the regimental surgeon.

When the 18th hussars embarked for England in 1807, one of the men of that regiment, who had been left behind in the King's Infirmary, joined at Rumford, with his scrotum very much enlarged and inflamed, his story being as follows:—Soon after leaving the infirmary, he jumped from a window, upon which he immediately perceived a swelling in the groin; on his landing in Liverpool, it became so large and painful that he could not walk, and was obliged to be forwarded to head-quarters in a cart. On handling the swelling, the surgeon of the regiment having perceived an unusual crepitus, as if air were diffused through the cellular substance, immediately wrote a state of the case to the late Mr. Obré, who was surgeon to the King's Infirmary, and in the mean time he used warm applications, purgatives, and low regimen. Mr. Obré, in reply, inclosed a paper that had been picked up in the ward of the infirmary in which this man lay, containing "a receipt for making a rupture," in which were directions to puncture the bag with a corking pin, and then, by means of a piece of a tobacco-pipe, to blow it up with air; and, if it were wished to produce a double rupture, the same thing was to be done on the other side, after which warm poultices were to be applied to take down the inflammation. The man strenuously denied ever having made use of any such means. By the applications directed, the swelling gradually subsided, but, while under treatment, he got, or pretended to get, a pain in the right hip and groin, and inability to move the limb. From this man's previous history, his new complaint was believed to be feigned. He was placed on low diet; the shower-bath was used along with purgatives, a mercurial course, repeated blisters and issues; but he would not yield.

The commanding officer of the regiment, disregarding the opinion of the surgeon and Dr. Warren, deputy inspector of hospitals, had this man brought forward and discharged, previous to the embarkation of the corps for the Peninsula, and was much displeased because the surgeon refused to sign a recommendatory certificate; by which refusal the reported man, who had been many years in the regiment, lost his pension. The same commanding officer, after his return from Corunna, met this man in London perfectly well, and following the laborious occupation of a porter. (*Dublin Hospital Reports.*)

---

### CHEMISTRY.

*Rheine, a peculiar Substance in Rhubarb.*—M. VAUDIN treated one part of rhubarb with eight parts of nitric acid at 36° (Baumé) with a gentle heat, and then reducing it to the consistence of a syrup, and diluting it with water, he observed that a peculiar substance was precipitated, which he has denominated Rheine, and which has the following properties when dried:—It is of a yellowish orange colour, without any peculiar smell; its taste is rather bitter, and it is almost entirely soluble in alcohol and ether: these solutions become yellow by acids, and of a rose-colour by alkalies. Rheine burns like other vegetable bodies, especially starch. Rhubarb treated directly by sulphuric ether yields a perfectly similar substance: this proves that the new substance exists already formed in the rhubarb, and that it is not acted upon by the nitric acid. (*Journ. de Chim. Med.*)

---

*On the Detection of Hydrocyanic Acid in the Bodies of Animals poisoned by it*, by MM. LASSAIGNE and LEURET.—The extreme delicacy of the test employed by M. Lassaigne to detect prussic acid in the bodies of poisoned animals,—namely, by means of the per-sulphate of iron, or the per-sulphate of copper, has been already pointed out. Being desirous of ascertaining after how long a period the poison might be discovered, MM. Lassaigne and Leuret made experiments in which animals were killed by hydrocyanic acid introduced into their food, or thrown into the stomach in a diluted state, the dose never surpassing more than the equivalent of five or six drops of the pure acid, or less than two drops: the animals were left after death for twenty-four hours in a chamber, and then buried in moist earth a foot and a half or two feet deep.

The disinterment took place after various periods, from fifteen days to a month, and the stomach and first portions of intestines being separated, were well divided and mixed with pure water, and distilled; a little sulphuric acid was added to the substances in the alembic, and the recipient was cooled by ice and water.

The products were rendered slightly alkaline, and then tested by the per-sulphate of iron or sulphate of copper, and a little excess of muriatic acid was poured upon the precipitates occasioned by these salts. No prussic acid could be discovered in animals, the viscera of which were in a state of complete putrefaction: it was found after intervals of two or three days, but never after a longer period than eight days.

From these experiments it appears—1. That prussic acid cannot be discovered in animals poisoned by small quantities of it, after exposition of these bodies for two or three days to the air. 2. That the disappearance of the poison in the viscera after a longer period than this is due to its decomposition, which is favoured by the contact of the putrescent animal matter. 3. That when it is necessary to examine a body judicially to ascertain the presence of this poison, it is important that it should be done as quickly as possible. (*Journal of Science.*)

---

*Sulphate of Quina.*—According to MM. Pelletier and Caventou, not less than 90,000 ounces of sulphate of quina was manufactured in 1826 in France; a quantity which could not have been administered to less than 1,444,000 persons. (*Jour. de Chimie. Med.*)

---

*Adulteration of Sulphate of Quina with Sugar.*—M. Winkler has found sulphate of quina, in commerce, adulterated with sugar. The quantity of adulteration was ascertained by dissolving the salt in water, precipitating the quina by carbonate of potass, filtering the liquid, evaporating to dryness, and digesting the residuum in alcohol. This fluid left the sulphate and carbonate of potash, but dissolved the sugar, and by evaporation the latter was obtained in its separate state.

M. Winkler has also met with benzoic acid adulterated with sulphate of lime in crystals. (*Bull. Univ.*)

---

*Change of Oil of Turpentine by Exposure to Air.*—Some oil of turpentine which had been exposed to air, was distilled by MM. Boissenot and Perset, who obtained a very acid liquid from it, which consisted of acetic acid, and a substance which crystallised upon cooling. These crystals were colourless and transparent, inodorous, insipid, very slightly soluble in cold water, but very soluble in hot water: they were similarly affected by alcohol and ether. Strong acids dissolved this substance, but it was not affected by alkalies. It was formed of oxygen, hydrogen, and carbon, and does not exist in oil of turpentine recently distilled. (*Jour. of Science.*)

---

*A Method of detecting minute Quantities of Opium in solution;* by R. Hare, M.D.—Through the discoveries of Sertuerner, it is now well known that opium contains an alkaline substance, called morphia, to which it owes its efficacy in promoting sleep, and relieving pain; also that this alkali is naturally in union with an acid called meconic, which produces a striking red colour with solutions of red oxyd of iron. Nevertheless, this property has not been proposed as a means of detecting opium; which has probably arisen from the circumstance that the meconate of iron does not precipitate. I have, however, contrived a method by which a quantity of opium not exceeding that contained in ten drops of laudanum may be detected in a half-gallon of water.

My process is founded on the property which meconic acid has of precipitating with lead. Hence, by adding a few drops of acetate of lead to any infusion containing any quantity of the drug in question, not more minute than the proportion above mentioned, an observable quantity of the meconate of lead falls down. The precipitation, where the quantity is small, may require from six to twelve hours, and may be facilitated by a very gentle stirring with a glass rod to detach the flocks from the sides of the recipient, which should be conical, so as to concentrate them during their descent. The meconate being thus collected at the bottom of the vessel, let about thirty drops of sulphuric acid be poured down on it by means of a glass tube. Let this be followed by as much of the red sulphate of iron. The sulphuric acid liberates the meconic acid, and thus enables it to produce, with the iron, the appropriate colour which demonstrates the presence of that acid, and consequently of opium. (*Philadelphia Journal.*)

---

### MISCELLANEOUS.

*On the Comparative Nutritive Properties of different kinds of Food.*—A very interesting Report on this subject was formerly presented to the French minister of the interior, by MM. Percy and Vauquelin, two members of the Institute, the accuracy of which may be depended on. It may, at this period of public distress, be valuable in those families where the best mode of supporting nature should be adopted at the least expence.

The result of their experiments is as follows:—In bread, every hundred pounds weight are found to contain eighty pounds of nutritious matter. Butcher's meat, averaging the various sorts, contains only thirty-five pounds in one hundred. Broad beans, eighty-nine. Pease, ninety-three. Lentils (a kind of half-pea, but little known in England,) ninety-four pounds in one hundred. Greens and turnips, which are the most aqueous of all the vegetables used for domestic purposes, furnish only eight pounds of solid nutritious substance in one hundred. Carrots, fourteen pounds. And, what is remarkable, as being in opposition to the hitherto acknowledged theory, one hundred pounds of potatoes only yield twenty-five pounds of substance, valuable as nutrition.

One pound of good bread is equal to two pounds and a half, or three pounds, of the best potatoes; and seventy-five pounds of bread, and thirty pounds of meat, are equal to three hundred pounds of potatoes. Or, to go more into detail, three-quarters of a pound of bread, and five ounces of meat, are equal to three pounds of potatoes; one pound of potatoes is equal to four pounds of cabbage, and three of turnips; but one pound of rice, broad beans, or French beans, is equal to three pounds of potatoes. (*Edinburgh New Philosophical Journal.*) All this cannot be true

---

*Coccus Cacti.*—This little insect, so valuable for yielding the cochineal dye and carmine, has of late years been introduced not only into the East Indies, but likewise into some of our West India islands. In St. Vincent's, the Rev. Lansdown Guilding, a distinguished naturalist, has established a nopalery (or cochineal nursery) in his own garden; and it is believed he has already sent specimens of the dried insect to the Society of Arts in London. The species of cactus, or nopal, planted by Mr. Guilding for the insects to feed and breed upon, is the C. cochinillifer of Linnæus, but not of Decandolle; which last is C. Tuna of Linnæus. In Mexico two varieties, or perhaps species, of the insect are bred: a superior kind called *fina*, and a common kind called *silvestre*. It is the latter only which we have yet acquired; but the East India Company having offered a large reward for the introduction into Bengal of the former, we may hope that this will soon be accomplished. The importance of the object will appear, when it is mentioned that the annual consumption of cochineal in Great Britain alone is estimated at 150,000 ℔., which is worth 275,000*l.* sterling. For about twelve years past, a few of the insects have been kept on cacti, in one of the hot-houses in the King's garden at Kew. Like the common coccus of our pine-stoves, the male is winged, and flies about; while the female is destitute of wings, and scarcely ever changes her place. A good representation of the cochineal cactus, with some of the insects upon it, has just been published by Professor Hooker, of Glasgow, in the Botanical Magazine; a work now conducted in a style of the first excellence under that gentleman's management. (*Ibid.*)

